# *DNMT3B* (rs2424913) polymorphism is associated with systemic lupus erythematosus alone and with co-existing periodontitis in a Brazilian population

**DOI:** 10.1590/1678-7757-2021-0567

**Published:** 2022-04-29

**Authors:** Larissa Nadine da Silva Dias, Marina de Castro Coêlho, Darlene Camati Persuhn, Isabella Lima Arrais Ribeiro, Eutilia Andrade Medeiros Freire, Naila Francis Paulo de Oliveira, Sabrina Garcia de Aquino

**Affiliations:** 1 Universidade Federal da Paraíba Centro de Ciências da Saúde Programa de Pós Graduação em Odontologia João Pessoa PB Brasil Universidade Federal da Paraíba - UFPB, Centro de Ciências da Saúde, Programa de Pós Graduação em Odontologia, João Pessoa, PB, Brasil.; 2 Universidade Federal da Paraíba Centro de Ciências Exatas e da Natureza Departamento de Biologia Molecular João Pessoa PB Brasil Universidade Federal da Paraíba - UFPB, Centro de Ciências Exatas e da Natureza, Departamento de Biologia Molecular, João Pessoa, PB, Brasil.; 3 Universidade Federal da Paraíba Centro de Ciências Médicas Departamento de Medicina Interna João Pessoa PB Brasil Universidade Federal da Paraíba - UFPB, Centro de Ciências Médicas, Departamento de Medicina Interna, João Pessoa, PB-Brasil.; 4 Universidade Federal da Paraíba Centro de Ciências da Saúde Departamento de Odontologia Clínica e Social João Pessoa PB Brasil Universidade Federal da Paraíba- UFPB, Centro de Ciências da Saúde, Departamento de Odontologia Clínica e Social, João Pessoa, PB, Brasil.

**Keywords:** Periodontitis, Systemic lupus erythematosus, Polymorphism, MTHFR, DNMT, Inflammation

## Abstract

The association between Periodontitis and Systemic Lupus Erythematosus (SLE) has been primarily based on their similar pathophysiology and both are associated with genetic polymorphisms. Objectives: To investigate an association between the methylation-related gene polymorphisms *DNMT3B* (rs2424913) and *MTHFR* (rs1801133) to Systemic Lupus Erythematosus (SLE) and Periodontitis. Methodology: In total, 196 individuals of all genders aged 24 to 60 years old were allocated into four groups based on their systemic and periodontal status, namely: Healthy control (n=60), periodontitis (n=51), SLE (n=47), and SLE + periodontitis (n=38). Individuals with SLE were stratified according to disease activity (SLEDAI) in inactive or active. We performed polymorphism analysis using PCR-RFLP with genomic DNA from mouthwash. We analyzed data using Fisher’s Exact, Chi-square test, and regression models. Results: Periodontal status were similar in subjects with periodontitis alone and combined with SLE. SLE patients with periodontitis had a longer SLE diagnosis than SLE only (p=0.001). For *DNMT3* B polymorphism, the periodontitis, SLE, and Inactive SLE + periodontitis groups showed a higher frequency of T allele and TT genotypes compared to healthy controls (p<0.05). Regression analyses showed that the TT genotype is a strong risk factor for periodontitis (OR=4.53; CI95%=1.13–18.05) and also for SLE without periodontitis (OR=11.57; CI95%=3.12–42.84) and SLE with periodontitis (OR=5.27; CI95%=1.25–22.11) when compared to control. Conclusion: SLE patients with periodontitis had a longer length of SLE diagnosis. The *DNMT3B* (rs2424913) polymorphism was associated with periodontitis and SLE alone or combined with periodontitis. Our study contributes to understanding the genetic mechanisms involved in periodontitis and SLE susceptibility.

## Introduction

The association between Periodontitis and Systemic Lupus Erythematosus (SLE) has been primarily based on their similar pathophysiology.^1^ Periodontitis is a chronic inflammatory disease, in which attachment loss and bone destruction around teeth result from an imbalance in the host immune response against a dysbiotic dental biofilm in susceptible individuals.^2^ SLE is a systemic autoimmune disease characterized by connective tissue destruction in multiple organs under abnormal T cell activity and high autoantibody levels.^3^

Increasing epidemiologic evidence has shown a high prevalence and an increased risk of periodontitis in SLE patients.^4 , 5^ Conversely, the literature shows controversial data concerning periodontal status in SLE patients, and some studies reported a milder,^6^ a similar,^7 - 9^ and a more severe form of periodontitis in SLE patients.^10^ Moreover, interventional studies have demonstrated the beneficial effect of periodontal therapy in reducing SLE disease activity^11^ and ameliorating the response to treatment. Indeed, few mechanistic studies^12^ elucidate the relationship between periodontitis and SLE; however, most of the underlying mechanisms remain unknown.^12^

Studies have reported genetic polymorphisms in both periodontitis^13^ and SLE^14^ in genes bonded to inflammatory pathways. Despite the evidence of epigenetic mechanisms involved in both diseases, epigenetic pathways related genes are, so far, neglected. Researchers have reported DNA hypermethylation and hypomethylation in a variety of genes in individuals with periodontitis and SLE.^15 , 16^ These epigenetic changes, in turn, can be caused by an increase or decrease of enzymes involved in epigenetic mechanisms such as methylenetetrahydrofolate (MTHFR) and DNA methyltransferase (DNMT), for example.

The enzyme encoded by *MTHFR* originates during folate metabolism the S-adenosyl methionine (SAM) radical (a methyl donor), whereas *DNMT* uses SAM as a substrate in the DNA methylation process.^17^ In particular, C677T *MTHFR* (rs1801133) polymorphism is related to a decreased enzyme activity of methylenetetrahydrofolate reductase, and -149C→T *DNMT3B* (rs2424913) polymorphism is related to an increased expression of *DNMT3B* . Changes in the activity or concentration of these enzymes can influence the DNA methylation profile, which can, in turn, lead to changes in gene expression. ^18 , 19^

Studies have shown the involvement of *MTHFR* or *DNMT3B* polymorphisms in inflammatory diseases.^20 , 21^ Recently, our group revealed the association of the 149C→T *DNMT3B* (rs2424913) polymorphism with periodontitis,^22^ but not for C677T *MTHFR* (rs1801133). Our study aimed to study the association of these polymorphisms in individuals with SLE, as well as in individuals with SLE and periodontitis.

## Methology

### Study design

This is a cross-sectional study comprising 196 individuals of all genders, aged 24 to 60 years old.

### Research ethics

This study followed the Helsinki Declaration and was approved by the Institutional Research and Ethics Committee at the Federal University of Paraiba (UFPB) (CAAE: 03948118.0.0000.5183). All subjects signed an informed consent form.

### Study local

The study was conducted at the dental (Department of Clinical and Social Dentistry) and medical (Center of Rheumatology at the Lauro Wanderley University Hospital) clinics of the Federal University of Paraíba, João Pessoa-PB, Brazil. The latter is a reference center for diagnostic and treatment of patients with SLE in the State of Paraíba. Individuals were selected to compose the study from new attendances and diagnosis realized during 2019.

### Sample size

Adopting a medium effect size of proportion (0.50) for interest outcome of our study (difference in genotypic profile between SLE with and without periodontitis), a minimal necessary sample of 47 individuals with the outcome of interest (SLE) was estimated, considering a study power of 80% and a confidence interval of 95%. The *a posteriori* sample size was evaluated based on effect sizes for comparisons between the different subject groups and resulted in a study with an inference power of 80.24%. These estimates were performed using the G*Power software (version 3.1.9.2, Faul F., Universität Kiel, Germany).

### SLE and periodontal assessment

*SLE status –* All patients with SLE fulfilled at least four of the 11 criteria for the diagnostic classification of SLE established by the American College of Rheumatology.^23^ This index comprises both clinical and laboratory characteristics to determine the activity of the disease. A rheumatologist classified in the first visit SLE activity into inactive (Systemic Lupus Erythematosus Disease Activity Index: SLEDAI <5) or active (SLEDAI ≥5) stages according to the guidelines of the American Academy of Rheumatology.^23^ SLEDAI aims to assess disease activity in the last 10 days, by clinical and laboratory criteria.^24^ The scale ranges from 0 to 105, with a grade being attributed to each clinical and laboratory domain evaluation. Moreover, medical history, including time since SLE diagnosis and medications protocol, were analyzed from the patient’s medical record.

*Periodontal status-* Periodontal diagnosis was assessed by a single examiner previously calibrated (κ=0.90) and included plaque index (PI), probing depth (PD), bleeding on probing (BOP), clinical attachment level (CAL) at six sites per tooth as well as the number of missing teeth (at least 15 remaining natural teeth). Periodontal assessment was based on the current Classiﬁcation of Periodontal and Peri-Implant Diseases and Conditions.^25^ The control group (periodontal health) consisted of CAL=0 and PD<3 mm and BOP in <10% of sites. The primary criteria considered for the diagnosis of periodontitis was interproximal clinical attachment loss at least of 3 mm detected in two or more nonadjacent interproximal sites and secondarily PD≥5mm (which corresponds to stages II, III, and IV).^25^ As that current periodontal inflammatory status does not influence the polymorphism analysis, SLE patients with stable periodontitis in a reduced periodontium according to the current Classification of Periodontitis^25^ were included in the SLE + periodontitis group.

According to systemic condition (systemic health or with SLE diagnosis) and periodontal status (absence or presence of periodontitis), a convenience sample was categorized into four groups as follows: healthy control (n=60), periodontitis (n=51), SLE (n=47) and SLE + periodontitis (individuals with SLE also diagnosed with periodontitis) (n=38). Demographic and medical history of participants were assessed using a questionnaire or the medical chart patient analysis. The exclusion criteria considered individuals under 18 years old, history of HIV or hepatitis, current diabetes, pregnancy, orthodontic treatment, cognitive disorders, smoking habits, and other autoimmune and chronic diseases (except Periodontitis and Systemic Lupus Erythematosus)

### Sample collection

Genomic DNA samples were obtained from buccal mucosa cells by a mouthwash. Briefly, the cells were harvested by a one-minute mouthwash with 3% dextrose solution (6 mL) followed by TNE addition (3 mL). The homogenates were centrifuged at 3000 rpm for 10 minutes, and pelleted cell samples were stored in lysis solution at -20°C until DNA extraction.^26^

### DNA extraction and genetic polymorphism analysis

Genomic DNA was purified of oral epithelial cells using 8 M ammonium acetate as previously described.^26^ The quantity and purity of DNA were determined on a Nanodrop spectrophotometer. The analysis of the single nucleotide polymorphisms (SNPs) C677T *MTHFR* (rs1801133) and -149C→T DNMT3B (rs2424913) was performed using the PCR-RFLP technique (polymerase chain reaction–restriction fragment length polymorphism), which consisted of DNA amplification by PCR and digestion of DNA fragments by a restriction enzyme. Restriction enzyme activity determined the presence or absence of the SNP. Briefly, PCR was performed using 100 ng of total DNA in a 15 μL final volume reaction containing 7.5 μL of GoTaq® G2 Hot Start Green Master Mix (Promega), 4.5 μL of DNA, and nuclease-free water in the presence of 1 μL of each primer (10 μM). Primer sequences, as well as both PCR and enzymatic digestion conditions, were used as previously described. PCR products were resolved by electrophoresis on 6% (w/v) polyacrylamide gels containing GelRed® (Biotium) or silver nitrate. The 677 CC/CT/TT and -149 CC/CT/TT genotypes were identified by their band pattern.^22^ SNPs were selected using the dbSNP database ( http://www.ncbi.nlm.nih.gov/projects/SNP/ ) and were chosen based on functional significance and its relationship to inflammatory diseases. Minor allele frequency according to 1000Genomes database is 0.24 for rs1801133 and 0.31 for rs2424913.

### Statistical analysis

The collected data were inserted in a Microsoft Excel platform and analyzed by descriptive and inferential statistic using the R Software (version 3.6.1). Demographic data were analyzed descriptively and the study groups ((1) control; (2) periodontitis; (3) SLE; (4) SLE+Periodontitis) were compared by One Way ANOVA with Tukey *post-hoc* test (for age, number of missing teeth, and CAL comparisons), by Chi-Square test with Yates correction (for gender comparisons), Student’s *t* -test (for SLE duration comparisons), by Chi-Square test (for SLE activity, Medication use, Periodontal status, Sites with periodontal pockets, and Probing depth comparisons), by Kruskal-Wallis test (for Bleeding sites comparisons) and Mann-Whitney U test (for Sites with periodontal pockets and Probing depth comparisons). Hardy-Weinberg equilibrium (HWE) was estimated for each polymorphism using the Chi-Square test. The Fisher’s Exact test and Chi-Square test with and without Yates’ correction were used to analyze possible associations among allele and genotypic frequencies, SLE and periodontitis. Binary logistic regression models were used to evaluate the variables associated to outcomes of interest. Five models were adjusted for regression analysis using the backward method: (Model 1): Periodontitis (1) × Control (0); (Model 2): SLE (1) × Control (0); (Model 3): SLE × Periodontitis (1) × Periodontitis (0); (Model 4): SLE with periodontitis (1) × Control; (Model 5): SLE with periodontitis (1) × periodontitis (0); (Model 6): SLE (1) × SLE with Periodontitis (0). In adjust of regression models, an univariate analysis was performed and, sequentially, the variables of interest that showed a significance level ≤ 0,20 were included in multiple models for adjustment by backward method, remaining in adjusted models for each outcome only variables with significance level of 5%. For all the analysis the significance level adopted was 5%.

## Results

### Demographic and periodontal conditions

Table 1 shows the demographic data and the clinical and periodontal status of the study population. The subjects in the control, Periodontitis, SLE, and SLE + Periodontitis groups had a similar mean age with no significant differences: Control (38.48±8.16 years old), Periodontitis (48.96±11.30 years old), SLE (33.17±8.95 years old), SLE + Periodontitis (38.87±10.03 years old); (p=0.110).

**Table 1:** Demographic data and clinical and periodontal status of the study population including the mean age, the mean time of SLE diagnosis (in years), disease activity (inactive or active), gender distribution, current use of drugs as well as periodontal status and the number of missing teeth are described as the mean ± SD or total number (n) and percentage (%); periodontal parameters (bleeding sites, sites with periodontal pockets, probing depth and clinical attachment level are described as median (p25,p75)

Variable	Control	Periodontitis	SLE	SLE + Periodontitis	p value
	**(n = 60)**	**(n = 51)**	**(n = 47)**	**(n = 38)**	
**Age (mean SD)**	38.48 (±8.16)	48.96 (±11.30)	33.17 (±8.95)	38.87 (±10.03)	0.110 I
**Gender n (%)**					
Female	47 (78.30%)	29 (56.90%)	43 (91.50%)	36 (94.70%)	<0.001 II
Male	13 (21.70%)	22 (43.10%)	4 (8.50%)	2 (5.30%)	
**SLE duration (years)**	-	-	5.21	10.34	0.001 III
**(mean SD)**			(5.17)	(7.81)	
**SLE activity**					
Inactive SLE n (%)	-	-	20 (42.60%)	24 (63.20%)	<0.001 IV
Active SLE n (%)	-		27 (57.40%)	14 (36.80%)	
Medication use					
Use of corticosteroid n (%)	-	-	18 (38.30%)	16 (42.10%)	0.086 IV
Use of immunosuppressive n (%)	-	-	30 (63.80%)	17 (44.70%)	0.825 IV
Use of hydroxychloroquine n (%)	-	-	42 (89.40%)	34 (89.50%)	0.987 IV
**Periodontal status**					
Stage II n (%)	-	25 (49.00%)	-	18 (47.40%)	0.877 IV
Stage III or IV n (%)	-	26 (51.00%)	-	20 (52.60%)	
Number of missing teeth (mean SD)	-	7.84 (5.84)	8.04 (5.15)	9.16 (4.54)	0.332 I
Bleeding sites (median (p25; p75))	-	41.00 a (24.00; 56.00)	3.00 b (1.00; 8.00)	19.50 c (7.00; 40.00)	<0.001 V
Sites with periodontal pockets (median (p25; p75))	-	25.00 (16.00; 46.00)	-	12.00 (6.00; 23.75)	0.002 VI
Probing depth (median (p25; p75))	-	7.00 (6.00; 8.00)	-	5.50 (5.00; 7.00)	0.032 VI
CAL (mean SD)	-	4.77 (+1.57)	-	5.14 (+1.42)	0.253 III

I One-way ANOVA (Tukey post-hoc test);

II Chi-Square test with Yates Correction;

III Student T-test;

IV Chi-Square test;

V Kruskal-Wallis test;

VI Mann-Whitney U test. Significance level=5%.

According to the medical records, patients with SLE without periodontitis showed a shorter time of SLE diagnosis (5.21±5.17 years), whereas patients from the SLE + Periodontitis group had a longer length of SLE diagnosis (10.34±7.81 years); (p=.001). The number of inactive cases (n=20; 42.6%) was lower than of active cases (n=27; 57.40%) in the SLE group (p<0.001). For the SLE + periodontitis group, 63.20% (n=24) of subjects had inactive SLE, while 36.80% (n=14) of individuals had active SLE (p<0.001). No difference occurred in the profile of medication used by SLE subjects and patients with SLE and periodontitis (p>0.05).

Periodontal status was similar in both the periodontitis and SLE + periodontitis groups, showing no significant differences (p=0.877). We diagnosed subjects from the periodontitis group as Stage II (n=25; 49.0 %) and Stage III/IV (n=26; 51.0%); individuals with SLE + periodontitis were classified as Stage II (n=18; 47.4%) and Stage III/IV (n=20; 52.6%). The number of missing teeth was similar in all groups: periodontitis (7.84±5.84), SLE (8.04±5.15) and SLE + periodontitis (9.16±5.54); (p=0.332).

### Genetic polymorphism

Table 2 and 3 shows the allelic and genotypic distributions of the study population. Genotypic frequencies are in accordance with HWE for both studied polymorphisms: *MTHFR* (rs1801133): Control (p=0.57), Periodontitis (p=0.13), SLE (p=0.25) and SLE + periodontitis (p=0.27), and *DNMT3B* (rs2424913): Control (p=0.54), Periodontitis (p=0.89), SLE (p=0.72) and SLE + periodontitis (p=0.80).

**Table 2:** Comparative analysis of allele frequencies of the studied polymorphisms (MTHFR and DNMT3B) among the control, periodontitis, SLE and SLE + periodontitis groups

Gene SNP	*MTHFR* C677T(rs1801133)		*DNMT3B* C46359T (rs2424913)	
Allelic frequency	C	T	C	T
**Control** ^1^ **n=60**	85 (70.80%)	35 (29.20%)	82 (68.30%)	38 (31.70%)
**Perio** ^2^ **n=51**	69(67.60%)	33(32.40%)	48(47.06%)	54(52.94%)
**p value^1.2^**	0.181		**0.011**	
**SLE** ^3^ **n=47**	70(74.50%)	24(25.50%)	37(39.36%)	57(60.64%)
**p value^1.3^**	0.565		**<0.001**	
**Inactive SLE** ^4^ **n=20**	28(70.00%)	12(30.00%)	13(32.50%)	27(67.50%)
**p value^1.4^**	0.897		**0.002**	
**Active SLE** ^5^ **n=27**	42(77.78%)	12(22.22%)	24(44.44%)	30(55.56%)
**p value^1.5^**	0.360		0.002	
**SLE + Perio** ^6^ **n=38**	51(67.10%)	25(32.90%)	41(53.95%)	35(46.05%)
**p value^1.6^**	0.529		0.138	
**Inactive SLE + Perio** ^7^ **n = 24**	32(66.67%)	16(33.33%)	24(50.00%)	24(50.00%)
**p value^1.7^**	0.810		**0.020**	
**Active SLE + Perio** ^8^ **n= 14**	19(67.86%)	09(32.14%)	17(60.71%)	11(39.29%)
**p value^1.8^**	0.773		0.370	

Chi-Square test and Chi-Square test with Yates’s correction. Significance level = 5%.

**Table 3:** Comparative analysis of genotype frequencies of the studied polymorphisms (MTHFR and DNMT3B) among the control, periodontitis, SLE and SLE + periodontitis groups

Gene SNP	*MTHFR* C677T (rs1801133)			*DNMT3B* C46359T (rs2424913)		
Genotype frequencies	CC	CT	TT	CC	CT	TT
**Control** ^1^	31	23	6	27	28	5
**n=60**	(51.70%)	(38.30%)	(10.00%)	(45.00%)	(46.70%)	(8.30%)
**Perio** ^2^	21	27	3	11	26	14
**n=51**	(41.20%)	(52.90%)	(5.90%)	(21.60%)	(51.00%)	(27.50%)
**p-value** ^1 , 2^		0.275			**0.005**	
**SLE** ^3^	27	16	4	8	21	18
**n=47**	(57.40%)	(34.00%)	(8.50%)	(17.00%)	(44.70%)	(38.30%)
**p-value** ^1 , 3^		0.842			**<0.001**	
**Inactive SLE** ^4^	10	8	2	1	11	8
**n=20**	(50.00%)	(40.00%)	(10.00%)	(5.00%)	(55.00%)	(40.00%)
**p-value** ^1 , 4^		0.991			**<0.001**	
**Active SLE** ^5^	17	8	2	7	10	10
**n=27**	(63.00%)	(29.60%)	(7.40%)	(26.00%)	(37.00%)	(37.00%)
**p-value** ^1 , 5^		0.618			**0.003**	
**SLE + Perio** ^6^	19	13	6	11	19	8
**n=38**	(50.00%)	(34.20%)	(15.80%)	(28.90%)	(50.00%)	(21.10%)
**p-value** ^1 , 6^		0.735			0.105	
**Inactive SLE** ^+^Perio^7^	11	10	3	7	10	7
**n=24**	(45.80%)	(41.70%)	(12.50%)	(29.20%)	(41.70%)	(29.20%)
**p-value** ^1 , 7^		0.876			**0.042**	
**Active SLE** ^+^Perio^8^	8	3	3	4	9	1
**n=14**	(57.20%)	(21.40%)	(21.40%)	(28.60%)	(64.30%)	(7.10%)
**p-value** ^1 , 8^		0.378			0.620	

Chi-Square test and Chi-Square test with Yates’s correction. Significance level = 5%.

For *MTHFR* , the C allele and genotypes CC and CT were the most common distributions in all groups, and we observed no significant differences (p>0.05).

For the *DNMT3B* polymorphism, the frequency of the T allele and the TT genotype respectively was higher in the periodontitis (52.9%; 27.5%) and in SLE (60.5%; 38.3%) groups compared to the control group (31.7%; 8.3%), even when the groups with inactive (67.5%; 40%) and active (55.5%; 37%) form of SLE were compared separately with the control group (p<0.05). When the comparison was made between the control (31.7%; 8.3%) and the SLE + periodontitis (46%; 21.1%) group, the difference was no longer observed (p>0.05). However, when stratifying the group according to the activity of SLE we detected a higher frequency of the T allele and the TT genotype in the SLE group with inactive form (50%; 29.2%) in comparison to the control group (p<0.05).

### Binary logistic regression analysis

Table 4 shows Binary Logistic regression (adjusted and non-adjusted) for different outcomes. We can evaluate that the TT allele it’s a strong risk factor for periodontitis compared to control (OR=4.53; CI95%= .13 – 18.05) and also for SLE without periodontitis compared to control (OR=11.57; CI95%= .12 – 42.84) and SLE with periodontitis (OR=5.27; CI95% = 1.25 – 22.11) compared to control. As expected, the chance of observing bleeding sites is 1.05 times smaller in people with SLE without periodontitis compared to people with SLE with periodontitis (OR: 0.95 [0.92-0.98]).

**Table 4:** Results for binary logistic regression (non-adjusted and adjusted) for different outcomes, considering gender, age, DNMT3B, bleeding sites, and sites with periodontal pocket

Outcomes	Variable	Categories	Non–adjusted			Adjusted		
			p value	OR	IC 95%	p value	OR	IC 95%
**Periodontitis x Control**	Gender	Female	-	1.00	-	-	-	-
		Male	0.017	2.74	1.20-6.27	-	-	-
	Age		<0.001	1.11	1.06-1.16	<0.001	1.11	1.05-1.16
	*DNMT3B*	CC	-	1.00	-	-	1.00	-
		CT	0.067	2.27	0.94-5.50	0.363	1.60	0.58-4.43
		TT	0.002	6.87	1.20-23.71	0.032	4.53	1.13-18.05
**SLE x Control**	Gender	Female	-	1.00	-	-	-	-
		Male	0.074	0.33	0.10-1.11	-	-	-
	Age		0.003	0.92	0.88-0.97	0.008	0.92	0.87-0.98
	*DNMT3B*	CC	-	1.00	-	-	1.00	-
		CT	0.061	2.53	0.95-6.68	0.094	2.37	0.86-6.53
		TT	<0.001	12.15	3.42-43.11	<0.001	11.57	3.12-42.84
**SLE x Periodontitis**	Gender	Female	-	1.00	-	-	1.00	-
		Male	<0.001	0.12	0.03-0.39	0.023	0.11	0.01-0.73
	Age		<0.001	0.85	0.79-0.91	0.003	0.82	0.72-0.93
	Bleeding sites		<0.001	0.92	0.89-0.95	<0.001	0.91	0.87-0.95
**SLE with periodontitis x Control**	Gender	Female		1.00	-	-	-	-
		Male	0.042	0.20	0.04-0.94	0.026	0.15	0.03-0.79
	Age		0.834	1.00	0.96-1.05			
	*DNMT3B*	CC		1.00	-	-	1.00	-
		CT	0.273	1.66	0.66-4.14	0.240	1.75	0.68-4.45
		TT	0.042	3.92	1.05-14.68	0.023	5.27	1.25-22.11
**SLE with Periodontitis x Periodontitis**	Gender	Female	-	1.00	-	-	-	-
		Male	0.001	0.07	0.01-0.33	-	-	-
	Age		<0.001	0.91	0.87-0.96	0.004	0.90	0.83-0.96
	Sites with periodontal pocket		0.047	0.96	0.93-0.98	0.024	0.95	0.92-0.99
**SLE x SLE with Periodontitis**	Bleeding sites		0.002	0.95	0.92-0.98	0.002	0.95	0.92-0.98

* Some variables were not evaluated for all groups and, for this reason, only those that were evaluated in the compared groups are shown for each outcome.

## Discussion

Considering the marked role of genetic, epigenetic, and environmental factors on the onset of both SLE and periodontitis, our study investigated for the first time the contribution of methylation-related gene polymorphisms to the clinical association between SLE and periodontitis. Here we identified that the polymorphism -149C→T *DNMT3B* (rs2424913) was bonded with both diseases. Indeed, demographic and clinical findings suggest that periodontitis develops similarly in SLE patients and that its establishment may be associated with the length of SLE diagnosis.

Indeed, we observed a lower prevalence of periodontitis in SLE patients (25.9%) in comparison with findings from other studies (42.59% to 88.53%).^10 , 27 , 28^ We can also attributed it to variations and a lack of clarity of periodontal case definitions used to diagnose periodontitis.^4 , 8^ Tooth loss is an important parameter that has been incorporated into the current classification of periodontal disease used in our study.^25 , 29^ In our data, the mean tooth loss was similar among systemically healthy and SLE subjects with periodontitis. To date, there is an emerging discussion regarding the inclusion of patients with extensive tooth loss and stable remaining teeth (with no current active periodontal disease) in periodontal medicine studies.^29^ Notably, we observed SLE patients with this clinical profile: tooth loss and signs of previous history of periodontitis, which were included in the SLE + periodontitis group. However, a longitudinal follow-up is important to accurately distinguish the causes of high tooth loss due to periodontal or other dental problems, including caries, in SLE patients.^30^

Regarding periodontal diagnosis, we observed a similar periodontal condition between the Periodontitis and SLE + periodontitis groups, as verified in other investigations,^8 , 9^ contrasting to a worse periodontal condition in SLE patients found by others.^10^ Moreover, SLE patients without periodontitis showed a protective factor in the number of bleeding sites in comparison to SLE patients with periodontitis, in agreement with a previous study.^31^ Intriguingly, patients with SLE + periodontitis had a smaller chance of observing sites with periodontal pockets when compared to patients with periodontitis only. These findings may be related to the chronic use of medications by SLE patients, which may affect the infectious and inflammatory processes. For instance, increased doses of immunosuppressive agents in nonresponding patients may possibly contribute to the control of periodontal inflammation.^32 , 33^ In contrast, prolonged use of corticosteroids at high doses has been associated with periodontal destruction in patients with SLE.^8^ Importantly, periodontitis was most frequent in active SLE patients with a longer length of SLE diagnosis (p=0.001) and, consequentially, under prolonged treatment. This finding highlights the importance of an early periodontal diagnosis to prevent the onset and progression of periodontal disease in patients with SLE.

The study of genetic polymorphisms is extremely important in gene mapping and the search for genes involved in common disorders, with important applicability for personalized medicine.^34^ In particular, *MTHFR* or *DNMT3B* polymorphisms have been associated with inflammatory diseases.^20 , 21^ The *MTHFR* and *DNMT* genes participate in the DNA methylation process, which in turn is an epigenetic marker that regulates gene expression. The methylenetetrahydrofolate reductase enzyme encoded by the *MTHFR* gene generates the methyl radical donor (SAM), and the DNA methyltransferase 3B, encoded by the *DNMT3B* gene, transfers the methyl radical from SAM to the DNA.^17^ In particular, the rs1801133 polymorphism is associated with decreased methylenetetrahydrofolate activity and consequent decrease in SAM levels, which may lead to DNA hypomethylation,^35 , 36^ while the rs2424913 polymorphism is associated with increased DNMT3B expression, which may lead to DNA hypermethylation.^37^ As previously mentioned, DNA methylation is an epigenetic marker that regulates gene expression, and generally, hypomethylation is associated with increased transcription and hypermethylation is associated with decreased transcription.^17^

In our study, we observed that individuals with periodontitis, SLE and inactive SLE + periodontitis showed a higher frequency of the T allele and the TT genotype for *DNMT3B.* The data on periodontitis confirm our previously results^22^ and for the first time we show an association between rs2424913 polymorphism and SLE and SLE concomitant with periodontitis. The TT genotype (no CT genotype) has been associated with an upregulation of DNMT3B and increased hypermethylation status.^37^ Hypermethylation has been reported in periodontitis in genes involved in prostaglandin synthesis, gene encoding receptors that recognize molecular patterns associated with pathogens, gene encoding pro-inflammatory cytokines and genes that regulate the translation process.^38^ In SLE, hypermethylation occurs in genes involved in: leukocyte activation, regulation of cell proliferation and death, control of granulocyte differentiation and positive regulation of metabolic processes.^39^ However, the relationship between hypermethylation at specific sites and the presence of the rs2424913 polymorphism in these populations remains to be clarified.

Intriguingly, the frequency of the T allele and TT genotype observed in Inactive SLE + periodontitis group was not observed in the Active SLE + periodontitis group. We may hypothesize that this can be explained by the smaller sample size in the Active SLE + periodontitis group or possibly by a different biological mechanism with the influence of other polymorphisms in patients with concomitant active form of SLE and periodontitis. Recently, a review emphasized that SLE is the most heterogeneous disease treated by physicians, making diagnosis and treatment a challenge.^40 , 41^ Other authors have argued that SLE is not just one disease, but many, based on different clinical presentations and their degrees of severity.^40 , 42^ Thus, based on the data from our study, we can speculate that the heterogeneity of the disease may also be related to different metabolic pathways, which in turn are determined by different polymorphisms. In fact, the authors state that genetics is not only involved in susceptibility to SLE but is also involved in the clinical course of the disease. Moreover, the literature shows that SLE may have a multifactorial etiology (environmental factors associated with genetic polymorphisms) or monogenic etiology (mutations).^42 - 44^

While data on polymorphisms related to SLE or periodontitis are relatively vast,^44 , 45^ to the best of our knowledge, few studies have evaluated genetic polymorphisms in a population with SLE + periodontitis. Kobayashi, et al.^10^ (2003) showed an association of the *FcgammaR* polymorphism with SLE + periodontitis in a Japanese population. In a Mendelian randomization study, the *IGF2R* polymorphism was associated with periodontitis and SLE in a European population.^12^ Both studies disregarded the degree of SLE severity, as in our study.

In contrast to the findings for *DNMT3B* (rs2424913), the C677T *MTHFR* polymorphism was not associated with periodontitis and/or SLE, as the C allele and the CC and CT genotypes were the most frequent genotypes in the entire population. Individuals who are homozygous for the wild-type C allele have normal enzyme activity, whereas heterozygotes retain 65% of wild-type MTHFR enzymatic activity.^35^ The data in relation to periodontitis confirm our results previously found^22^ and in relation to SLE are consistent with Salimi, et al.^44^ (2017) that found no association too. However, TT genotype that have only 30% of the MTHFR enzymatic activity^35^ was alreary found in non-syndromic cleft lip, lichen planus, cardiovascular risk, and squamous cell carcinoma.^46 - 48^

Our study focused on methylation-related gene polymorphisms associated with inflammatory diseases, an investigation which studies had never been focused on before in diagnosed SLE patients with periodontitis. However, several target genes still need to be analyzed. The limitation of our study relates to its cross-sectional design, small sample size due to the well-defined inclusion criteria as well as a narrow sample collection period. Therefore, other studies should further explore this data in larger populations with different ethnic backgrounds which could provide definitive conclusions.

## Conclusion

This study demonstrates the association of the polymorphism -149C→T *DNMT3B* (rs2424913) with the occurrence of SLE and concomitant SLE + periodontitis in individuals with the inactive form of SLE. Moreover, SLE patients with periodontitis had a longer length of SLE. Genetic studies provide a biological database that can be used as tools for precision medicine, enabling a more accurate line of diagnosis, treatment and prophylaxis.

## References

[1] Sete MR, Figueredo CM, Sztajnbok F. Periodontitis and systemic lupus erythematosus. Rev Bras Reumatol. 2016;56(2):165-70. doi: 10.1016/j.rbre.2015.09.001 10.1016/j.rbre.2015.09.001 27267530

[2] Tonetti MS, Greenwell H, Kornman KS. Staging and grading of periodontitis: framework and proposal of a new classification and case definition. J Clin Periodontol. 2018;45(Suppl 20):149-61. doi: 10.1002/JPER.18-0006 10.1111/jcpe.12945 29926495

[3] Tsokos GC. Systemic lupus erythematosus. N Engl J Med. 2011;365(22):2110-21. doi: 10.1111/jcpe.12945 10.1056/NEJMra1100359 22129255

[4] Rutter-Locher Z, Smith TO, Giles I, Sofat N. Association between systemic lupus erythematosus and periodontitis: a systematic review and meta-analysis. Front Immunol. 2017;17;8:1295. doi: 10.3389/fimmu.2017.01295 10.3389/fimmu.2017.01295 PMC5650969 29089946

[5] Graves DT, Corrêa JD, Silva TA. The oral microbiota is modified by systemic diseases. J Dent Res. 2018;98(2):148-56. doi: 10.1177/0022034518805739 10.1177/0022034518805739 PMC6761737 30359170

[6] Mutlu S, Richards A, Maddison P, Scully C. Gingival and periodontal health in systemic lupus erythematosus. Community Dent Oral Epidemiol. 1993;21(3):158-61. doi: 10.1111/j.1600-0528.1993.tb00742.x 10.1111/j.1600-0528.1993.tb00742.x 8348790

[7] Calderaro DC, Ferreira GA, Corrêa JD, Mendonça SM, Silva TA, Costa FO, et al. Is chronic periodontitis premature in systemic lupus erythematosus patients? Clin Rheumatol. 2017;36(3):713-8. doi: 10.1007/s10067-016-3385-8 10.1007/s10067-016-3385-8 27557901

[8] Mendonça SM, Corrêa JD, Souza AF, Travassos DV, Calderaro DC, Rocha NP, et al. Immunological signatures in saliva of systemic lupus erythematosus patients: influence of periodontal condition. Clin Exp Rheumatol. 2019;37(2):208-14. 30148445

[9] Rezaei M, Bayani M, Tasorian B, Mahdian S. The comparison of visfatin levels of gingival crevicular fluid in systemic lupus erythematosus and chronic periodontitis patients with healthy subjects. Clin Rheumatol. 2019;38(11):3139-43. doi: 10.1007/s10067-019-04708-w 10.1007/s10067-019-04708-w 31372850

[10] Kobayashi T, Ito S, Yamamoto K, Hasegawa H, Sugita N, Kuroda T, et al. Risk of periodontitis in systemic lupus erythematosus is associated with fcγ receptor polymorphisms. J Periodontol. 2003;74(3):378-84. doi: 10.1902/jop.2003.74.3.378 10.1902/jop.2003.74.3.378 12710759

[11] Fabbri C, Fuller R, Bonfá E, Borba EF. Periodontitis treatment improves systemic lupus erythematosus response to immunosuppressive therapy. Clin Rheumatol. 2014;33(4):505-9. doi: 10.1007/s10067-013-2473-2 10.1007/s10067-013-2473-2 24415114

[12] Bae SC, Lee YH. Causal association between periodontitis and risk of rheumatoid arthritis and systemic lupus erythematosus: a Mendelian randomization. Z Rheumatol. 2020;79(9):929-36. doi: 10.1007/s00393-019-00742-w 10.1007/s00393-019-00742-w 31965238

[13] Silva MK, Carvalho AC, Alves EH, Silva FR, Pessoa LD, Vasconcelos DF. Genetic factors and the risk of periodontitis development: findings from a systematic review composed of 13 studies of meta-analysis with 71,531 participants. Int J Dent. 2017;2017:1914073. doi: 10.1155/2017/1914073 10.1155/2017/1914073 PMC5424192 28529526

[14] Teruel M, Alarcón-Riquelme ME. The genetic basis of systemic lupus erythematosus: what are the risk factors and what have we learned. J Autoimmun. 2016;74:161-75. doi: 10.1016/j.jaut.2016.08.001 10.1016/j.jaut.2016.08.001 27522116

[15] Hedrich CM, Mäbert K, Rauen T, Tsokos GC. DNA methylation in systemic lupus erythematosus. Epigenomics 2017;9(4):505-25. doi: 10.2217/epi-2016-0096 10.2217/epi-2016-0096 PMC6040049 27885845

[16] Almiñana-Pastor PJ, Boronat-Catalá M, Micó-Martinez P, Bellot-Arcís C, Lopez-Roldan A, Alpiste-Illueca FM. Epigenetics and periodontics: a systematic review. Med Oral Patol Oral Cir Bucal. 2019;24(5):e659-72. doi: 10.4317/medoral.23008 10.4317/medoral.23008 PMC6764711 31433392

[17] Weber M, Hellmann I, Stadler MB, Ramos L, Pääbo S, Rebhan M, et al. Distribution, silencing potential and evolutionary impact of promoter DNA methylation in the human genome. Nat Genet. 2007;39(4):457-66. doi: 10.1038/ng1990 10.1038/ng1990 17334365

[18] Nash AJ, Mandaviya PR, Dib MJ, Uitterlinden AG, Van Meurs J, Heil SG, et al. Interaction between plasma homocysteine and the MTHFR c.677C > T polymorphism is associated with site-specific changes in DNA methylation in humans. FASEB J. 2019;33(1):833-43. doi: 10.1096/fj.201800400R 10.1096/fj.201800400R 30080444

[19] Naghibalhossaini F, Mokarram P, Khalilia E, Naghibalhossaini S. DNMT3b -149C/T promoter variants and methylation of colorectal cancer-associated genes. Cancer Biomarkers. 2015;15(3):227-33. doi: 10.3233/CBM-150463 10.3233/CBM-150463 PMC12964684 25769449

[20] Dedoussis GV, Panagiotakos DB, Pitsavos C, Chrysohoou C, Skoumas J, Choumerianou D, et al. An association between the methylenetetrahydrofolate reductase (MTHFR) C677T mutation and inflammation markers related to cardiovascular disease. Int J Cardiol 2005;100(3):409-14. doi: 10.1016/j.ijcard.2004.08.038 10.1016/j.ijcard.2004.08.038 15837084

[21] Cai TT, Zhang J, Wang X, Song RH, Qin Q, Muhali FS, et al. Gene-gene and gene-sex epistatic interactions of DNMT1, DNMT3A and DNMT3B in autoimmune thyroid disease. Endocr J. 2016;63(7):643-53. doi: 10.1507/endocrj.EJ15-0596 10.1507/endocrj.EJ15-0596 27237591

[22] Coêlho MC, Queiroz IC, Viana JM Filho, Aquino SG, Persuhn DC, Oliveira NF. miR-9-1 gene methylation and DNMT3B (rs2424913) polymorphism may contribute to periodontitis. J Appl Oral Sci. 2020;28:1-11. doi: 10.1590/1678-7757-2019-0583 10.1590/1678-7757-2019-0583 PMC7137733 32267380

[23] Hochberg MC. Updating the American College of Rheumatology revised criteria for the classification of systemic lupus erythematosus. Arthritis Rheum. 1997;40(9). doi: 10.1002/art.1780400928. 10.1002/art.1780400928 9324032

[24] Touma Z, Urowitz MB, Gladman DD. Systemic lupus erythematosus disease activity index 2000 responder index-50 website. J Rheumatol 2002;40(5):733. doi: 10.3899/jrheum.130030 10.3899/jrheum.130030 23637378

[25] Caton JG, Armitage G, Berglundh T, Chapple IL, Jepsen S, Kornman KS, et al. A new classification scheme for periodontal and peri-implant diseases and conditions – Introduction and key changes from the 1999 classification. J Clin Periodontol. 2018;45(Suppl 20):S1-S8. doi: 10.1111/jcpe.12935 10.1111/jcpe.12935 29926489

[26] Aidar M, Line SR. A simple and cost-effective protocol for DNA isolation from buccal epithelial cells. Braz Dent J. 2007;18(2):148-52. doi: 10.1590/s0103-64402007000200012 10.1590/s0103-64402007000200012 17982556

[27] Gofur NR, Nurdiana N, Kalim H, Handono K. Periodontitis is associated with disease severity and anti-double stranded DNA antibody and interferon-gamma levels in patients with systemic lupus erythematosus. J Taibah Univ Med Sci. 2019;14(6):560-5. doi: 10.1016/j.jtumed.2019.09.005 10.1016/j.jtumed.2019.09.005 PMC6940663 31908645

[28] Zhang Q, Zhang X, Feng G, Fu T, Yin R, Zhang L, et al. Periodontal disease in chinese patients with systemic lupus erythematosus. Rheumatol Int. 2017;37(8):1373-9. doi: 10.1007/s00296-017-3759-5 10.1007/s00296-017-3759-5 28631047

[29] Beck JD, Papapanou PN, Philips KH, Offenbacher S. Periodontal medicine: 100 years of progress. J Dent Res 2019;98(10):1053-62. doi: 10.1177/0022034519846113 10.1177/0022034519846113 31429666

[30] Benli M, Batool F, Stutz C, Petit C, Jung S, Huck O. Orofacial manifestations and dental management of systemic lupus erythematosus: a review. Oral Dis. 2021;27(2):151-67. doi: 10.1111/odi.13271 10.1111/odi.13271 31886584

[31] Pessoa L, Aleti G, Choudhury S, Nguyen D, Yaskell T, Zhang Y, et al. Host-microbial interactions in systemic lupus erythematosus and periodontitis. Front Immunol. 2019;10. doi: 10.3389/fimmu.2019.02602 10.3389/fimmu.2019.02602 PMC6861327 31781106

[32] Al-Mutairi KD, Al-Zahrani MS, Bahlas SM, Kayal RA, Zawawi KH. Periodontal findings in systemic lupus erythematosus patients and healthy controls. Saudi Med J 2015;36(4):463-8. doi: 10.15537/smj.2015.4.10746 10.15537/smj.2015.4.10746 PMC4404481 25828284

[33] Fanouriakis A, Kostopoulou M, Alunno A, Aringer M, Bajema I, Boletis JN, et al. 2019 Update of the EULAR recommendations for the management of systemic lupus erythematosus. Ann Rheum Dis. 2019;78(6):736-45. doi: 10.1136/annrheumdis-2019-215089 10.1136/annrheumdis-2019-215089 30926722

[34] Horton RH, Lucassen AM. Recent developments in genetic / genomic medicine. Clin Sci. 2019;133(5):697-708. doi: 10.1042/CS20180436 10.1042/CS20180436 PMC6399103 30837331

[35] Fang JY, Xiao SD. Folic acid, polymorphism of methyl-group metabolism genes, and DNA methylation in relation to GI carcinogenesis. J Gastroenterol. 2003;38(9):821-9. doi: 10.1007/s00535-003-1207-7 10.1007/s00535-003-1207-7 14564626

[36] Nash AJ, Mandaviya PR, Dib MJ, Uitterlinden AG, van Meurs J, Heil SG, et al. Interaction between plasma homocysteine and the MTHFR c.677C>T polymorphism is associated with site-specific changes in DNA methylation in humans. FASEB J. 2019;33(1):833-43. doi: 10.1096/fj.201800400R 10.1096/fj.201800400R 30080444

[37] Naghibalhossaini F, Mokarram P, Khalili E, Naghibalhossaini S. DNMT3b -149C/T promoter variants and methylation of colorectal cancer-associated genes. Cancer Biomark. 2015;15(3):227-33. doi: 10.3233/CBM-150463 10.3233/CBM-150463 PMC12964684 25769449

[38] Jurdziński KT, Potempa J, Grabiec AM. Epigenetic regulation of inflammation in periodontitis: cellular mechanisms and therapeutic potential. Clin Epigenetics. 2020;12(1):186. doi: 10.1186/s13148-020-00982-7 10.1186/s13148-020-00982-7 PMC7706209 33256844

[39] Teruel M, Sawalha AH. Epigenetic variability in systemic lupus erythematosus: what we learned from genome-wide dna methylation studies. Curr Rheumatol Rep. 2017;19(6):32. doi: 10.1007/s11926-017-0657-5 10.1007/s11926-017-0657-5 PMC5819620 28470479

[40] Dörner T, Furie R. Novel paradigms in systemic lupus erythematosus. Lancet 2019;393(10188):2344-58. doi: 10.1016/S0140-6736(19)30546-X 10.1016/S0140-6736(19)30546-X 31180031

[41] Rivas-Larrauri F, Yamazaki-Nakashimada MA. Lupus eritematoso sistémico: ¿es una sola enfermedad? Reumatol Clin. 2016;12(5):274-81. doi: 10.1016/j.reuma.2016.01.005 10.1016/j.reuma.2016.01.005 26922326

[42] Javinani A, Ashraf-Ganjouei A, Aslani S, Jamshidi A, Mahmoudi M. Exploring the etiopathogenesis of systemic lupus erythematosus: a genetic perspective. Immunogenetics 2019;71(4):283-97. doi: 10.1007/s00251-019-01103-2 10.1007/s00251-019-01103-2 30671674

[43] Omarjee O, Picard C, Frachette C, Moreews M, Rieux-Laucat F, Soulas-Sprauel P, et al. Monogenic lupus: dissecting heterogeneity. Autoimmun Rev. 2019;18(10):102361. doi: 10.1016/j.autrev.2019.102361 10.1016/j.autrev.2019.102361 31401343

[44] Salimi S, Keshavarzi F, Mohammadpour-Gharehbagh A, Moodi M, Mousavi M, Karimian M, et al. Polymorphisms of the folate metabolizing enzymes: association with SLE susceptibility and in silico analysis. Gene 2017;637:161-72. doi: 10.1016/j.gene.2017.09.037 10.1016/j.gene.2017.09.037 28943344

[45] Heidari Z, Moudi B, Mahmoudzadeh-Sagheb H. Immunomodulatory factors gene polymorphisms in chronic periodontitis: an overview. BMC Oral Health. 2019;19(1):1-15. doi: 10.1186/s12903-019-0715-7 10.1186/s12903-019-0715-7 PMC6373099 30755190

[46] Amooee A, Dastgheib SA, Niktabar SM, Noorishadkam M, Lookzadeh MH, Mirjalili SR, et al. Association of Fetal MTHFR 677C > t polymorphism with non-syndromic cleft lip with or without palate risk: a systematic review and meta-analysis. Fetal Pediatr Pathol. 2021;40(4):337-53. doi: 10.1080/15513815.2019.1707918 10.1080/15513815.2019.1707918 31880477

[47] Rashed L, Abdel Hay R, AlKaffas M, Ali S, Kadry D, Abdallah S. Studying the association between methylenetetrahydrofolate reductase (MTHFR) 677 gene polymorphism, cardiovascular risk and lichen planus. J Oral Pathol Med. 2017;46(10):1023-9. doi: 10.1111/jop.12588 10.1111/jop.12588 28463405

[48] Ge W, Jiao Y, Chang L. The association between MTHFR gene polymorphisms (C677T, A1298C) and oral squamous cell carcinoma: a systematic review and meta-analysis. PLoS One. 2018;13(8):e0202959. doi: 10.1371/journal.pone.0202959 10.1371/journal.pone.0202959 PMC6108503 30142181

